# Encountering competitors reduces clutch size and increases offspring size in a parasitoid with female–female fighting

**DOI:** 10.1098/rspb.2007.0867

**Published:** 2007-08-14

**Authors:** Marlène Goubault, Alexandra F.S Mack, Ian C.W Hardy

**Affiliations:** School of Biosciences, University of NottinghamSutton Bonington Campus, Loughborough, Leicestershire LE12 5RD, UK

**Keywords:** clutch size, game-theory, contests, intergenerational fitness

## Abstract

Understanding the size of clutches produced by only one parent may require a game-theoretic approach: clutch size may affect offspring fitness in terms of future competitive ability. If larger clutches generate smaller offspring and larger adults are more successful in acquiring and retaining resources, clutch size optima should be reduced when the probability of future competitive encounters is higher. We test this using *Goniozus nephantidis*, a gregarious parasitoid wasp in which the assumption of size-dependent resource acquisition is met via female–female contests for hosts. As predicted, smaller clutches are produced by mothers experiencing competition, due to fewer eggs being matured and to a reduced proportion of matured eggs being laid. As assumed, smaller clutches generate fewer but larger offspring. We believe this is the first direct evidence for pre-ovipositional and game-theoretic clutch size adjustment in response to an intergenerational fitness effect when clutches are produced by a single individual.

## 1. Introduction

The fitness of individuals which develop in discrete clutches is usually affected by the size of the clutch, due to limits on the amount of total resource available. When decisions are made by just one parent, the problem of how many eggs to lay can be addressed by static optimality models based on the marginal value theorem: for a given amount of resource, larger clutches usually generate more offspring in total but with lower *per capita* fitness and the optimal clutch size decreases from the ‘Lack solution’, which maximizes fitness per clutch, as trade-offs between current and future reproduction increase (e.g. Skinner 1985; Wilson & Lessels 1994).

Optimal clutch size decisions are more complex when multiple, competing, conspecific parents lay eggs into the same ‘clutch’ (i.e. group of eggs with multiple maternity): decisions have to be made in anticipation of, and/or in response to, the decisions of competitors and a game-theoretic modelling approach is required. Under such intraspecific competition, the evolutionarily stable number of eggs each parent should lay is predicted to decrease as the number of parents contributing to a clutch increases (Parker & Courtney 1984; Parker & Begon 1986; Ives 1989; Strand & Godfray 1989; Ruxton & Broom 2002). This result generally applies when resource competition between developing offspring is of the scramble type (when clutch mates obtain roughly similar resource quantities) but under contest competition (when one clutch member despotically excludes others from the resource) parents should each contribute a greater number of eggs to increase the probability of one of their own offspring winning the competition (Ives 1989).

While it is clear that a game-theoretic approach is required for considering situations in which multiple, and ultimately competing, parents contribute eggs to each clutch, a complete understanding of the decisions of parents that are the only contributors of eggs to their clutches may also require game-theory. This is because clutch size may affect offspring fitness in terms of the offspring's future ability to compete with conspecifics that have developed in other clutches. Thus, a parent producing a clutch should take into account the sizes of the clutches produced simultaneously by conspecifics elsewhere and the likelihood that their own offspring will competitively encounter individuals developing from these clutches. Assuming that larger clutches generate smaller offspring and that larger adults are more successful than smaller adults in acquiring and retaining resources via dyadic contests, game-theoretic modelling predicts that optimal clutch size is reduced in comparison with the prediction from a static optimality approach (Mesterton-Gibbons & Hardy 2004). This is an example of clutch size optima being influenced by an intergenerational fitness effect (Mangel *et al*. 1994).

Here we test the prediction that clutch size will be reduced in anticipation of a more competitive future environment, and the associated assumption that larger adults will mature from such reduced clutches. We use *Goniozus nephantidis*, the wasp species with the clutch size biology and competitive behaviour that stimulated the theoretical development (Petersen & Hardy 1996; Mesterton-Gibbons & Hardy 2004).

### (a) *Goniozus nephantidis:* clutch size and competition

*Goniozus nephantidis* (Hymenoptera: Bethylidae) is a gregarious parasitoid of lepidopteran larvae. On encountering a host, the female stings and paralyses it. Eggs are laid onto the host approximately 24 hours later, with clutch size ranging up to 18 eggs and positively correlated with host size (approx. 5–90 mg; Hardy *et al*. 1992). Manipulation experiments indicate that, for a given host size (30–40 mg), smaller individuals tend to emerge from larger clutches (Hardy *et al*. 1992). Competition between bethylid siblings feeding on the same host is of the scramble type (Mayhew & Hardy 1998).

As well as taking a relatively long time to lay eggs on paralysed hosts, females remain with their offspring until these have reached an advanced stage of larval development (i.e. approx. 8–10 days; Goubault *et al*. in press). Both before and after oviposition, the guarded host may be encountered by conspecific females and direct, aggressive and decisive intruder–owner contests usually ensue, with the loser being excluded from the vicinity of the host (Petersen & Hardy 1996; Humphries *et al*. 2006). Intruders that take over the host usually destroy any eggs already present and lay their own clutch (Goubault *et al*. in press): each host thus ultimately supports the offspring of just one female. While contest outcomes are influenced by the value that the competing females place on the host resource (Stokkebo & Hardy 2000; Humphries *et al*. 2006; Goubault *et al*. in press), owner–intruder asymmetries in body size are well established as a major determinant of contest outcomes: larger individuals tend to win (Petersen & Hardy 1996; Humphries *et al*. 2006; Goubault *et al*. in press, see also Goubault *et al*. 2006).

Since clutch size normally affects body size which in turn affects contest ability, anticipation of the competitive environment of offspring should affect maternal clutch size decisions in *G. nephantidis*. There are two, mutually non-exclusive, mechanisms by which the effect could operate: an evolutionary response to the long-term mean probability of contest interactions and a facultative response to factors related to shorter-term variations in this probability (Godfray *et al*. 1991; Wilson 1994; Creighton 2005). Because *G. nephantidis* naturally attacks an outbreak pest with highly variable population densities (Cock & Perera 1987), we expect the facultative mechanism to operate. Our experiments test the prediction of reduced clutch size by exposing host-guarding ‘owners’, that have not yet laid eggs, to different numbers of intruders. An experience of intrusion is expected to serve as a proxy for population density, and thus the likelihood of future competition.

## 2. Material and methods

*Goniozus nephantidis* were reared on larvae of *Corcyra cephalonica* (Stainton) (Lepidoptera: Pyralidae). The host and parasitoid strains were the same as used in the study by Goubault *et al*. (in press) and rearing procedures were as described by Stokkebo & Hardy (2000). Culturing and experiments were carried out in a climate room at 27°C, 12 L : 12 D and a high relative humidity maintained by a water bath (Goubault *et al*. in press).

Females designated as owners were individually placed in stoppered glass tubes and provided with one host larva, which the female paralysed. ‘Intruder’ females were isolated in empty glass tubes. Owners and intruders which encountered each other in subsequent experiments were of the same age (2–4 days), not siblings and had been weighed to an accuracy of 0.01 mg. Females used in experiments had not previously encountered a host since maturity and were naive with respect to host resource contests. Individuals were distinguished by marking with a dot of red or yellow acrylic paint on the dorsal surface of their thorax (Petersen & Hardy 1996).

Contest treatments were carried out using the three-chambered contest block developed by Petersen & Hardy (1996). The owner female plus paralysed host was placed in the central chamber (diameter 1.8 cm, depth 0.6 cm) of the block with a barrier isolating the chamber from the lateral chambers. The competition intensity was manipulated by varying the content of one of the lateral chambers, which was either empty (control treatment) or contained an intruder female, and by varying the number of times an owner encountered an intruder (see below). After 10 min, the barrier was withdrawn such that females could move between the chambers but were confined within the block (Petersen & Hardy 1996). Events within the central chamber were filmed from above with a video camera and the number and type of encounters (non-aggressive, chases, bites, attacks with the stinger and fights) between owner and intruder females were recorded. After 30 min, the barrier was reclosed to isolate the central chamber and, in treatments with intruders, the intruder removed. There were three intruder treatments: owners were sequentially exposed to one, two or four individual intruders. As with the one-intruder treatment, owners in two- or four-intruder treatments were exposed to intruders after being separated for 10 min and the intruder was removed 30 min later, with the procedure repeated for each new intruder.

After experimental treatment, owners were individually placed with their host in a stoppered glass tube and the number of eggs they subsequently laid (usually 24–48 hours after contest treatment) was noted on daily inspection. Some females were then dissected as soon as oviposition was observed to have occurred (i.e. within 24 hours of oviposition) in order to measure the number of mature eggs remaining in their ovaries (egg load). The number of adult offspring emerging from each brood was noted and female progeny were weighed to an accuracy of 0.01 mg.

In a first series of experimental replicates (sample sizes for 0, 1, 2 or 4 intruders were *N*=23, 17, 20 and 11, respectively), we used hosts weighing 30–40 mg (mean±s.e.: 35.15±0.35 mg) as this is the (deliberately narrow) range used in several prior studies (Hardy *et al*. 1992; Petersen & Hardy 1996; Stokkebo & Hardy 2000; Goubault *et al*. in press). No effects of intruder presence on clutch size or of clutch size on offspring size were detected (see §3). We then modified the experiment by using smaller hosts in the range 20–30 mg (mean±s.e.: 25.67±0.26 mg; sample sizes for 0, 1, 2 or 4 intruders were *N*=22, 21, 22 and 21, respectively). Further, in the second series of replicates, owners and intruders remained confined within the central chamber during the observation periods, which ensured that owners were fully exposed to any intruders present: owners and intruders were initially separated by the barrier bisecting the chamber, while in the withdrawn position the barrier was flushed with the edges of the chamber. Effects of intruder presence on clutch size and of clutch size on offspring size were detected from the second set of replicates. We refer to these separate sets of data as ‘big host’ and ‘small host’ experiments.

### (a) Statistical analyses

Data were analysed using generalized linear modelling within the Genstat statistical package (v. 8, VSN International, Hemel Hempstead, UK). We followed backward stepwise procedures to obtain parsimonious ‘minimum adequate’ models (e.g. Crawley 1993; Humphries *et al*. 2006). Log-linear analyses were used to explore the influences of host weight, maternal weight, competitor presence and agonistic encounters on the number of eggs matured and on clutch size, and to evaluate the influence of clutch size, host weight and competition on the number of offspring maturing from a clutch. Logistic analyses were used to identify factors influencing the proportion of a female's egg load laid and relationships between the proportion of eggs surviving to adulthood and clutch size, host weight and competition. In logistic and log-linear analyses, the scale parameter was estimated empirically to match the assumed and actual distributions of residuals and significance was thus assessed using *F*-ratio tests (Crawley 1993). Standard (Gaussian) analysis of covariance was used to test the effects of clutch size, host weight and competition on the mean weight of emerging daughters. All significance testing was two-tailed.

In the small host experiments, clutch size was highly correlated to host weight, maternal weight and the presence of competitors (see §3). We therefore explored, in both datasets, the effect of clutch size separately to that of host weight and competitor's presence in the analyses because the simultaneous inclusion of highly mutually correlated explanatory variables in a model can lead to interpretational problems due to collinearity (Grafen & Hails 2002; Quinn & Keough 2002).

## 3. Results

### (a) Big host

The number of eggs laid by owners was not influenced by host weight (*F*_1,70_=2.31, *p*=0.13), owner weight (*F*_1,70_=1.38, *p*=0.25) or the number of intruders present (*F*_3,70_=0.31, *p*=0.82; figure 1*b*). As the number of intruders did not affect clutch size decisions, intruders were treated as a dichotomous variable (present or absent) in the following analyses. The total number of eggs that owners matured (egg load) was not influenced by host weight (*F*_1,70_=0.27, *p*=0.61), owner weight (*F*_1,70_=3.64, *p*=0.06) or the presence of intruders (*F*_1,70_=0.42, *p*=0.52). The proportion of these mature eggs that were laid onto the host was not affected by host weight (*F*_1,70_=1.60, *p*=0.21), owner weight (*F*_1,70_=1.80, *p*=0.19) or intruder presence (*F*_1,70_=0.75, *p*=0.39). The proportion of eggs reaching adulthood was not influenced by the initial clutch size (*F*_1,70_=0.73, *p*=0.40), host weight (*F*_1,70_=0.20, *p*=0.65) or the presence of intruders (*F*_1,70_=1.64, *p*=0.21). The number of offspring eventually emerging as adults increased with clutch size (*F*_1,69_=166.00, *p*<0.001) but was not affected by host weight or the mothers' experience of intruders (*F*_1,70_=0.66, *p*=0.42 and *F*_1,70_=0.46, *p*=0.50, respectively; figure 1*d*). Finally, the mean weight of daughters was uninfluenced by the initial clutch size (*F*_1,70_=1.95, *p*=0.17; figure 1*f*), host weight (*F*_1,70_=1.39, *p*=0.24) or the presence of intruders (*F*_1,70_=0.16, *p*=0.69; figure 1*h*).

### (b) Small host

The number of eggs laid by owners increased with host weight (*F*_1,80_=6.57, *p*=0.013; figure 1*a*) and owner weight (*F*_1,80_=17.48, *p*<0.001) and was influenced by the number of intruders (*F*_3,80_=4.63, *p*=0.005; figure 1*a*) such that larger clutches were laid when intruders were absent. Clutch sizes were similar when one, two or four intruders were encountered (*F*_2,82_=1.07, *p*=0.35, following aggregation of factor levels; Crawley 1993, p. 190). Females that encountered intruders laid clutches of around two eggs (approx. 18%) smaller than females that did not experience competition (mean±s.e.: intruders present=9.10±0.47 and intruders absent=7.50±0.22). As the presence, rather than the number, of intruders influenced clutch size, intruder presence was subsequently treated as a dichotomous variable. When intruders were present, clutch size was uninfluenced by the occurrence of physically close owner–intruder encounters, or by the total number of agonistic interactions (*F*_1,63_=2.55, *p*=0.12 and *F*_1,63_=0.86, *p*=0.36, respectively). Clutch sizes produced by owners experiencing competition were also unaffected by the mean weight of the intruders encountered (*F*_1,63_=3.13, *p*=0.08).

The total number of eggs that owners matured was reduced in the presence of intruders (sample sizes for 0, 1, 2 or 4 intruders: *N*=18, 15, 17 and 13, respectively; *F*_1,60_=5.70, *p*=0.02; figure 2*a*) but increased with owner weight (*F*_1,60_=20.91, *p*<0.001; figure 2*a*). The increase in the number of mature eggs with increase in host weight was marginally non-significant (*F*_1,60_=3.89, *p*=0.054). The proportion of a female's mature eggs that were laid onto the host decreased with the presence of competitors (*F*_1,61_=4.06, *p*=0.049; figure 2*b*) but was not influenced by the weight of the owner (*F*_1,61_=0.08, *p*=0.77) or of the host (*F*_1,61_=0.04, *p*=0.85). The egg-to-adult survivorship (i.e. proportion of eggs reaching adulthood) increased with clutch size (*F*_1,84_=5.79, *p*=0.018) and with host weight (*F*_1,84_=8.31, *p*=0.005) but was not affected by maternal experience of intruders (*F*_1,84_=1.09, *p*=0.30). As expected therefore, the number of offspring emerging increased with clutch size (*F*_1,84_=78.44, *p*<0.001) and host weight (*F*_1,83_=13.08, *p*<0.001; figure 1*c*). Owing to the reduction in clutch size, the number of emerging offspring was lower when mothers experienced competition (mean±s.e.: no intruders, 7.18±0.65; intruders, 5.39±0.30; *F*_1,83_=5.34, *p*=0.023; figure 1*c*). The average weight of emerging daughters was influenced by the initial size of the clutch they developed from (figure 1*e*): smaller clutches produced larger females (*F*_1,83_=26.68, *p*<0.001) in a curvilinear relationship (quadratic term: *F*_1,83_=5.09, *p*=0.03). As females experiencing competition laid fewer eggs, they obtained larger daughters (*F*_1,84_=10.32, *p*=0.002; figure 1*g*); this effect was independent of the exact weight of small hosts (*F*_1,83_=2.53, *p*=0.12).

## 4. Discussion

Static optimality models of clutch size have been widely tested by empirical studies on, for instance, birds, beetles and parasitoid wasps, with at least partial or qualitative support (e.g. Lessells 1986; Godfray *et al*. 1991; Stearns 1992; Wilson 1994; Zaviezo & Mills 2000; Bezemer & Mills 2003). Clutch size responses predicted under the game-theoretic scenario of multiple mothers contributing eggs to a single clutch have also been tested. Among birds, clutch sizes are adjusted according to the probability of egg laying by multiple conspecifics (Power *et al*. 1989; Lyon 1998; Ruxton & Broom 2002; see also Hauber 2003 for analogous interspecific effects). Among beetles, *Callosobruchus maculatus* clutch size decisions may be independent of the probability of eggs being laid by conspecific females in the future (superparasitism) but responsive to the number of eggs already laid by other females (Wilson 1994); both facets of multiple maternity are expected to affect the intensity of inter-offspring competition during development. Among parasitoids, there is good evidence for contrasting clutch size responses according to the form of larval competition: when multiple maternity is more likely, the clutch size of individual mothers increases in solitary species in which only one offspring may complete development from each clutch (contest competition) and decreases in gregarious species in which many offspring may complete development in a clutch (scramble competition; Rosenheim & Hongkham 1996; Visser 1996; Goubault *et al*. 2005; Ito & Yamada 2005; Pexton & Mayhew 2005).

In contrast, the (game-theoretic) influence on clutch size of the future competitive environment of offspring maturing from clutches produced by single females has received very little empirical attention. Among birds, there is field evidence for the importance of the size of maturing great tit (*Parus major*) offspring relative to the size of their competitors, which suggests a possible effect on clutch size decisions (Both *et al*. 1999). More direct evidence is available for beetles: in *Nicrophorus orbicollis*, a burying beetle, adults compete directly for carcass (host) resources and larger contestants are advantaged. Clutches are subsequently produced by a single pair of parents, and clutch (brood) size is then manipulated via infanticide during brood development. Larger broods develop on larger carcasses and, for a given quantity of resource, smaller offspring are produced from larger broods (Creighton 2005). Creighton (2005) showed that beetles kept as immatures at high density produced fewer and larger offspring on carcasses of standard weight than those that had been kept at low density.

The data we present here are the first to explore the predicted effect in a parasitoid wasp. Our study organism, *G. nephantidis*, is the species the model was formulated to match (Mesterton-Gibbons & Hardy 2004). When large hosts were provided, we found no effect on clutch size of exposure to intruders, but there was also no relationship between clutch size and offspring size on large hosts. Given that a crucial assumption is violated, we would not predict that clutch size should be reduced when competition is more frequent (indeed, the model developed by Mesterton-Gibbons & Hardy (2004) predicts smaller effects on clutch size optima when offspring body size is less affected by changes in clutch size). The lack of relationship between clutch size and the exact weight of large hosts may be due to the both absolutely (10 mg) and relatively (33%) small variation in the weight of ‘large’ hosts, as the relationship has previously been found to be strong (explaining 52% of the variance) when larger ranges of host weights are presented (Hardy *et al*. 1992). Further, the range of clutch sizes laid on big hosts corresponds to the range on similar sized hosts observed previously (Hardy *et al*. 1992), given the large sample size in the current study. When small hosts were provided, we found that larger offspring develop from smaller clutches, matching an important assumption of the theory, and that smaller clutches were laid by mothers exposed to intruders, matching the prediction. Although the absolute variation in the weight of ‘small’ hosts was the same as for ‘large’ hosts, the relative variation (50%) was larger, which could explain why there was a relationship between exact host weight and clutch size. As above, the range of clutch sizes we observed on small hosts corresponds with the previously observed range on hosts in this weight range, given the larger sample size of the present study. The size of females developing from small hosts was generally greater than that of females developing from large hosts; this is unexpected given that Hardy *et al*. (1992) found that larger females developed on larger hosts. Our current data suggest that host quality and host size may not be simply correlated, possibly due to differences in nutritional factors (e.g. Häckermann *et al*. 2007).

We further found that females produce smaller clutches by both maturing fewer eggs and by laying lower proportions of the eggs that they have matured. Prior work has shown that *G. nephantidis* females mature eggs after emergence as adults (synovigeny), that egg maturation may be enhanced by possession of a host, especially a larger host, and that some mature eggs may remain unlaid during the oviposition of a given clutch (Stokkebo & Hardy 2000; Humphries *et al*. 2006; Goubault *et al*. in press). It is possible that the finding that females experiencing intruders lay lower proportions of their mature eggs is generated by smaller clutches being laid earlier than larger clutches and these females thus having more time to mature additional eggs before dissection: this currently seems unlikely as informal observations suggest no relationship between the timing of oviposition and clutch size (I.C.W. Hardy, personal observation), despite expectation (Petersen & Hardy 1996). The mechanisms by which *G. nephantidis* females adjust clutch size contrast with those reported for *N. orbicollis*, in which brood size adjustment occurs after eggs are laid (Creighton 2005). We conclude that *G. nephantidis* females make (game-theoretic) clutch size decisions based on the competitive environment likely to be experienced by their mature offspring (as predicted by Petersen & Hardy (1996) and Mesterton-Gibbons & Hardy (2004)).

Our results specifically support prior suggestions that the occurrence of contests between adult females for hosts contributes towards explaining the observation that *G. nephantidis* lays clutches smaller than the Lack solution calculated using a static optimality approach (considering only trade-offs between the number and *per capita* fitness of offspring generated by scramble-type sibling competition during development; Hardy *et al*. 1992; Petersen & Hardy 1996). Static optimality type trade-offs between current and future reproduction (e.g. Skinner 1985; Wilson & Lessels 1994) are also likely to contribute to the discrepancy because *G. nephantidis* is synovigenic and thus appears to be iteroparous rather than semelparous (Stokkebo & Hardy 2000; Humphries *et al*. 2006; Goubault *et al*. in press). While our results demonstrate a capacity for facultative adjustment of clutch size according to short-term variation in the expected future environment, *G. nephantidis* clutch size could also be evolving according to the longer term average intensity of female–female competition (as indicated by field evidence for *N. orbicollis*; Creighton 2005).

More generally, our results constitute evidence that clutch size optima are affected by intergenerational fitness effects and that maternal responses are phenotypically plastic (Mousseau & Dingle 1991; Mangel *et al*. 1994; Wilson & Lessels 1994). In this case, it appears that an enhanced probability of offspring experiencing future resource contests, which will have size-difference-dependent outcomes, selects for reduced clutch size and consequently larger offspring.

## Figures and Tables

**Figure 1:**
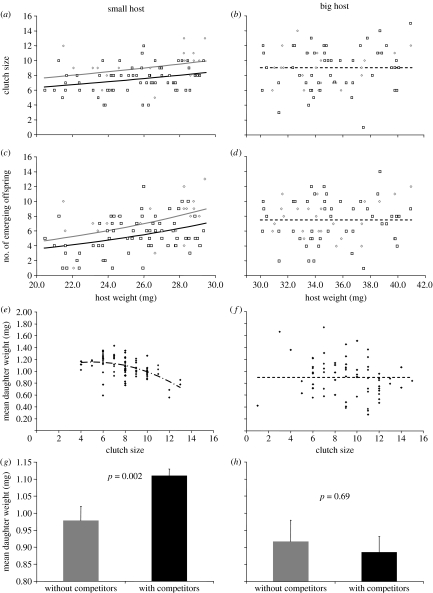
(*a*,*b*) Effects of host weight and competitor presence on clutch size and (*c,d*) subsequent consequences in terms of number and (*e*–*h*) weight of emerging offspring. (*a*, *c*, *e*, *g*) Relationships for small hosts (20–30 mg) are all stastically significant; (*b*, *d*, *f*, *h*) relationships for large hosts (30–41 mg) are not. Open diamonds, without competitors; open squares, with competitors; filled diamonds, with and without competitors; grey solid line, regression without competitors; black solid line, regression with competitors; dot-dashed line, regression with and without competitors; dashed line, mean.

**Figure 2:**
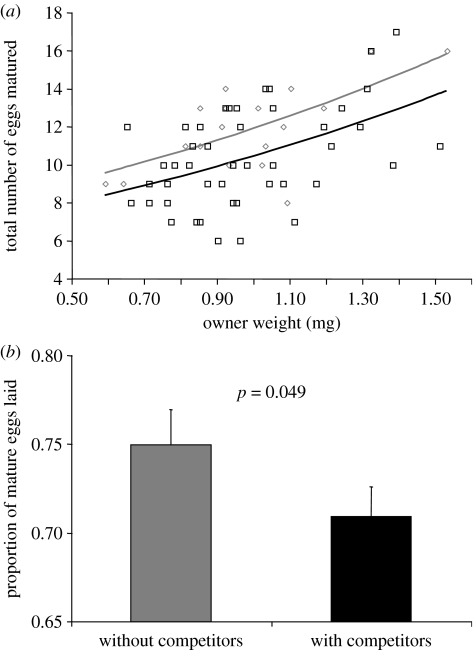
(*a*) Effects of owner weight and competitor presence on the total number of eggs matured by owners and (*b*) the effect of competitor presence on the proportion of these mature eggs that are laid. Open diamonds, without competitors; open squares, with competitors; grey solid line, regression with competitors; black solid line, regression with competitors.
